# Nephrotoxicity of HAART

**DOI:** 10.1155/2011/562790

**Published:** 2011-08-15

**Authors:** Robert Kalyesubula, Mark A. Perazella

**Affiliations:** ^1^Department of Medicine, School of Medicine, Makerere University College of Health Sciences, P.O. Box 7072, Kampal, Uganda; ^2^Section of Nephrology, Yale School of Medicine, BB 114, 330 Cedar Street, New Haven, CT 06520-8029, USA

## Abstract

Highly active antiretroviral therapy (HAART) and other medical therapies for HIV-related infections have been associated with toxicities. Antiretroviral therapy can contribute to renal dysfunction directly by inducing acute tubular necrosis, acute interstitial nephritis, crystal nephropathy, and renal tubular disorders or indirectly via drug interactions. With the increase in HAART use, clinicians must screen patients for the development of kidney disease especially if the regimen employed increases risk of kidney injury. It is also important that patients with chronic kidney disease (CKD) are not denied the best combinations, especially since most drugs can be adjusted based on the estimated GFR. Early detection of risk factors, systematic screening for chronic causes of CKD, and appropriate referrals for kidney disease management should be advocated for improved patient care. The interaction between immunosuppressive therapy and HAART in patients with kidney transplants and the recent endorsement of tenofovir/emtricitabine by the Centers for Disease Control (CDC) for preexposure prophylaxis bring a new dimension for nephrotoxicity vigilance. This paper summarizes the common antiretroviral drugs associated with nephrotoxicity with particular emphasis on tenofovir and protease inhibitors, their risk factors, and management as well as prevention strategies.

## 1. Introduction

Highly active antiretroviral therapy (HAART) consisting of at least three drugs active against HIV infection has revolutionized the management of HIV-AIDS. This has been reflected in the reductions in morbidity and mortality across the globe [1–3]. However, use of antiretroviral drugs has been associated with a number of toxicities, including those affecting the kidney [4]. The kidney plays a major role in the metabolism and excretion of antiretroviral drugs and this makes it vulnerable to various types of injuries from some of these agents, including acute kidney injury (AKI), tubulopathies, chronic kidney disease (CKD), and end-stage renal disease requiring renal replacement therapy. As the population of HIV-infected patients ages and remains on HAART for longer periods of time, age-, HIV- and HAART-related metabolic disorders are increasingly being encountered by clinicians looking after these patients.

This paper reviews recent advances on the HAART-related nephrotoxicity, with a particular emphasis on early recognition and management of patients who may be at increased risk.

## 2. Epidemiology of Nephropathy in the HIV-Infected Population since the HAART Era

Nephropathy in HIV can be caused by both HIV-related and non-HIV-related pathologies. Non-HIV related causes include hypertension, diabetes mellitus, atherosclerosis, drugs, primary and secondary nephropathies, as well as other infections [5].

HIV can cause direct injury to the kidneys as manifested by HIV-associated nephropathy (HIVAN). This entity was described before the era of HAART but continues to be a significant problem despite the advent of HAART [5–7]. HIVAN is the third leading cause of ESRD in African Americans who are also 18 times more likely to progress to ESRD than their white American counterparts [8]. A few years ago, HIVAN was initially considered to be genetically linked to a variation in the MYH9 locus of chromosome 22, which is found in 60% of African Americans and in less than 4% of Europeans [9]. However, recent researchers have noted that the MYH9 gene is located next to the APOL-1 gene which is more significantly associated with ESRD than all previously reported variations in MYH9 gene [10]. In less developed countries, patients often present late to medical attention and may have HIVAN; however, this renal lesion can develop in patients on HAART due to poor medication adherence.

Other forms of HIV-related nephropathies like HIVICK (HIV immune-complex kidney disease), HIV thrombotic microangiopathy, as well as kidney disease associated with opportunistic infections such as cytomegalovirus, mycobacterium, cryptosporidium and malignancies such as lymphoma and Kaposi's sarcoma are described [11–14]. Hepatitis B and hepatitis C infections have an increased prevalence in the HIV-positive population and cause various glomerular lesions. They also merit special mention because of the complexity of diagnosis and management of renal disease in the setting of combined HIV-HCV infection as well as its increased mortality risks [15–17]. Additionally, HAART and drugs used to treat opportunistic infections may cause renal disease. Thus, the vast etiologic spectrum of renal disease in HIV-infected patients is daunting, and HAART nephrotoxicity is a diagnosis of exclusion.

## 3. Epidemiology of HAART-Associated Nephrotoxicity

AKI that develops in the setting of HIV infection typically occurs with severe opportunistic infections, rather than as a sole consequence of direct toxicity of antiretrovirals. However, antiretroviral nephrotoxic effects accounted for 14% of late-onset AKI episodes, occurring after 3 months of initiating HAART [18]. AKI in hospitalized HAART naïve-HIV-1-infected patients is associated with a 6-fold higher risk of in-hospital mortality [19]. In the post-HAART era, HIV-infected patients with AKI still have an increased risk of in-hospital mortality, and these episodes of AKI seem more frequent in the first year of therapy [20] probably due late presentation of patients and severe immunosupression with concurrent infections at the time of admission. 

HAART has also been associated with CKD. The major drugs implicated in this include indinavir, atazanavir, and tenofovir [21]. The Development of Antiretroviral Therapy in Africa (DART) trial examined 3,316 symptomatic ART-naive adults from Uganda and Zimbabwe with CD4 < 200 cells/mm^3^ who were initiated on HAART with zidovudine-lamivudine plus tenofovir (74%), nevirapine (16%), or abacavir (9%). The study concluded that severe kidney dysfunction (<30 mL/min as estimated by the Cockcroft-Gault formula) occurred in only 2.7% of patients on all regimens and kidney disease contributed to death in a minority of patients, which was generally related to concurrent disease [22]. The major limitation was that renal tubular function was not assessed. 

Although a low incidence (0.3 to 2%) is noted [23], tenofovir (TDF) is the drug most often associated with Fanconi syndrome (FS) [24], which carries the potential consequences of calcium and phosphorus dysregulation and osteomalacia [25, 26]. In a meta-analysis of 17 studies (including 9 randomized, controlled trials) examining TDF safety, a significantly greater loss of kidney function among the TDF recipients, compared with control subjects (mean difference in calculated creatinine clearance, 3.92 mL/min; 95% confidence interval [CI], 2.13–5.70 mL/min), as well as a greater AKI risk (risk difference, 0.7%; 95% CI, 0.2–1.2), was noted [27].

## 4. Risk Factors Associated with Drug-Induced Nephrotoxicicity

Risk factors for nephrotoxicity are numerous and depend on underlying patient characteristics as well as the drug regimen under consideration. Traditional risk factors for kidney disease like hypertension, diabetes mellitus, and use of other nephrotoxic agents remain significant concerns in HIV patients on HAART [5, 28]. A number of observational studies have documented TDF-associated nephrotoxicity following its widespread use in patients with multiple comorbid conditions [22]. TDF-induced renal toxicity is more likely to occur in HIV patients with preexisting kidney disease or poorly controlled HIV disease with longer overall antiviral treatment duration, older age, elevated baseline creatinine concentration, female gender, African American ethnicity, CD4 nadir <200 cells/mm^3^, and concomitant administration of other nephrotoxic drugs [29, 30]. Combined therapy with TDF and protease inhibitors such as ritonavir appears to increase renal toxicity [31]. Conversely, HAART may increase the risk of hypertension, diabetes mellitus, and other metabolic complications creating a vicious cycle.

In a study by Wyatt et al., the major risk factors for AKI and associated mortality included severe immunosupression (CD4 count, <200 cells/mm^3^) and opportunistic infections [19]. Dehydration, alkaline urine, and a previous history of nephrolithiasis appear to be risk factors for atazanavir-associated kidney stones [32]. 

The risk factors for hyperlactemia (lactate > 2 mmol/L with or without acidosis) which is common with “d-drugs” like stavudine (d4T) and didanosine (ddI) include extended duration of treatment, old age, female gender, pregnancy, hypertriglyceridemia, obesity, hepatitis C infection, impaired kidney function, treatment with ribavirin, and alcohol use [33, 34].

## 5. Clinical and Pathological Presentation of HAART-Related Kidney Disorders

Clinically, HAART causes various kidney syndromes including various electrolyte and acid-base disorders, AKI, lactic acidosis, and chronic kidney disease. These injuries occur via multiple mechanisms, including direct tubular toxicity, allergic reactions, and precipitation of insoluble drug crystals within renal tubular lumens [35]. 

There are more than 20 drugs available on the market today for HIV treatment with the first-line therapies varying across the globe. Most of the first-line therapies include nucleos(t)ide reverse transcriptase inhibitors (NRTI) with either a protease inhibitor (PI) or a nonnucleoside reverse inhibitor (NNRTI).  Table 1 notes the preferred first-line ARV regimens by the WHO [36].

However, it is important to note that the choice of the nucleoside backbone depends on the patient's clinical and virological profile. For example, a baseline viral load >100,000 copies/mL makes a tenofovir-based regimen preferable over abacavir [37]. Patients with baseline kidney dysfunction may also benefit from the tenofovir-sparing regimen when such resources are available. However, the optimal glomerular filtration rate at which such a decision should be made is largely unknown. On the other hand, patients with HIV/HBV coinfection will require tenofovir plus lamivudine or emtricitabine as the backbone [36]. In such patients, the GFR should be estimated and the HAART dose should be adjusted accordingly. Of note, nucleoside-sparing regimens should be used with caution in patients with protease-resistant HIV viral isolates [38]. 

## 6. Acute Kidney Injury Related to Medications

Studies of AKI occurring in HIV-infected individuals have demonstrated that medications commonly employed to the treatment of HIV-related infections are important causes of kidney injury including ATN. Aminoglycoside antibiotics, pentamidine, acyclovir, foscarnet, amphotericin, tenofovir, adefovir, and cidofovir have all been associated with ATN in HIV-infected patients [39]. 

The possibility of rhabdomyolysis with pigment-related kidney injury should be considered in patients with HIV who develop AKI, particularly if they are being treated with zidovudine, didanosine, or integrase inhibitors [40–42]. In one renal biopsy series of European patients with HIV, approximately 10% of AKI cases were attributed to myoglobinuric pigment nephropathy [43].

Tenofovir, which is commonly used in combination with emtricitabine (FTC) as Truvada or as a single pill containing efavirenz/emtricitabine/tenofovir disoproxil fumarate (Atripla) [46], is widely prescribed and is an integral part of each of the four “preferred” regimens for treatment of HIV-1 in antiretroviral-naive adults and adolescents [47]. This popularity has largely been attributed to its convenient dosing schedule, antiviral efficacy, and relatively favourable side-effect profile, making it one of the most widely prescribed antiretroviral drugs for the treatment of HIV-1 [48]. The TDF/FTC combination is rapidly becoming popular even in resource-limited settings, especially with efforts to phase out the more toxic stavudine. TDF/FTC use is also likely to increase for post-HIV exposure prophylaxis and as part of treatment of choice for HIV patients coinfected with hepatitis B virus [36, 49]. The recent preexposure trial and interim guidelines by the CDC on the use of TDF/FTC in men who have sex with men make this drug even more important [50, 51]. 

**Figure 1:**
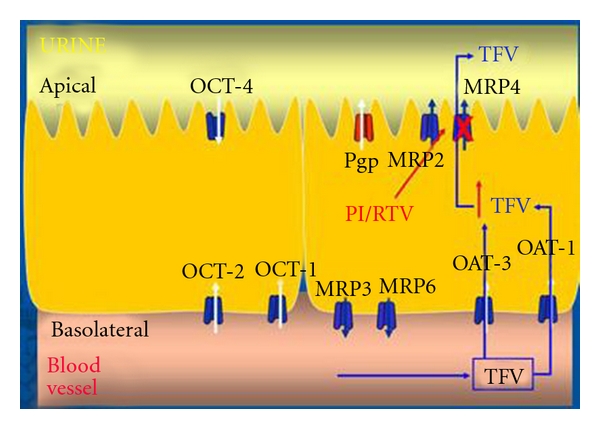
Tenofovir is predominantly eliminated via a combination of glomerular filtration and active tubular secretion. It enters into the kidney cell from the basolateral side via organic anion transporters, OAT-1 and OAT-3 [44], and leaves either via P glycoprotein, MRP2, and/or MRP4 [45]. Inhibition of MRP4 by PI/RTV leads to increased intracellular tenofovir levels which may increase its nephrotoxic effects. OAT: organic anion transporter; MRP: multidrug resistant protein; PI/RTV: ritonavir-boosted protease inhibitor; TFV: tenofovir.

Ritonavir-boosted PIs may have an increased propensity of causing renal injury. Approximately 70% of the published cases of TDF-induced nephrotoxic effects are observed with concomitant use of low-dose ritonavir. An interaction between lopinavir-ritonavir combination therapy and TDF, which manifests as a decrease in the renal clearance of TDF, has been identified [52]. TDF is actively taken up into the proximal tubules and secreted into the lumen via multidrug resistance-associated protein-4 [45]. Inhibition of MRP4 by PI/RTV leads to increased intracellular tenofovir levels that may increase its nephrotoxicity effects [45, 53] (see detailed mechanism in Figure 1).

Postmarketing safety data covering 455,392 person-years of TDF exposure showed serious renal adverse events in only 0.5% of patients and graded elevations in serum creatinine in 2.2% of patients (Nelson, MR). With time, TDF has been linked to the development of proximal tubular dysfunction including Fanconi syndrome (FS), AKI, nephrogenic diabetes insipidus (NDI), and severe hypokalemia [24, 31, 54–56]. Lamivudine, stavudine, abacavir, and didanosine have also been implicated in case reports of FS and NDI [30, 57, 58]. Fanconi syndrome caused by tenofovir-induced nephrotoxicity is characterized by generalized proximal tubular dysfunction resulting in one or more of the following: bicarbonaturia, glucosuria (with normal blood sugar), phosphaturia, uricosuria, aminoaciduria, and tubular proteinuria. It is hypothesized that this toxicity is the result of mitochondrial DNA depletion or direct tubular cytotoxicity similar to that associated with the use of adefovir and cidofovir [31, 59–61]. Most of these adverse reactions can be reversed with discontinuation of the drug, although some will not be completely reversed [62]. A recent study by the Columbia University group demonstrated that TDF nephrotoxicity is manifested as toxic acute tubular necrosis targeting proximal tubules (including FS in some cases) and manifests distinctive light microscopic and ultrastructural features of mitochondrial injury [63]. While all patients recovered kidney function, including discontinuation of dialysis, nearly half were left with some level of CKD. One limitation when assessing nephrotoxicity of TDF is the relatively short “96-week” followup in clinical trials, as Fanconi syndrome may occur after a relatively long time of tenofovir treatment.

In a forty-eight-week multicenter randomized trial comparing abacavir/lamivudine to TDF/FTC for adverse renal effects, efficacy, and safety in HAART-naïve patients (ASSERT study), there was no difference in estimated glomerular filtration rate between the arms [64]. However, increases in markers of tubular dysfunction were observed in the TDF/FTC arm, the long-term consequence of which is unclear. A significant difference in efficacy that favored TDF/FTC was also observed [64]. Abacavir/lamuvudine is an alternative combination to TDF/FTC.

The clinical implications of TDF use are unclear, but clinicians should routinely evaluate for FS and other nephrotoxicity in patients on this drug. In general, the beneficial immunological and virological responses gained with TDF largely outweigh potential renal toxicity.

Acute interstitial nephritis (AIN) has been described with indinavir, abacavir, ritonavir, and atazanavir [65–67]. In addition to ARVs, other drugs used for prophylaxis and treatment of opportunistic infections in HIV-AIDS patients, such as trimethoprim/sulfamethoxazole, amphotericin B, acyclovir, and antituberculous drugs like rifampicin, streptomycin, and pyrazinamide have been associated with AKI. Nonsteroidal anti-inflammatory drugs (NSAIDs), trimethoprim/sulfamethoxazole, and rifampin may cause acute interstitial nephritis in HIV-infected patients [68]. NSAIDS may also promote prerenal azotemia in patients with true or effective intravascular volume depletion. Abacavir causes renal toxicity as part of the systemic clinical syndrome of abacavir hypersensitivity, which can be avoided by the HLA-B* 5701 screening [69]. AIN often resolves with drug discontinuation, but steroid therapy in severe biopsy-proven cases may be beneficial when employed early.

In clinical trials, AKI has been reported to occur in 1% of patients assigned to etravirine [70]. In two randomized trials (DUET-1 and DUET-2) examining the efficacy and safety of etravirine in treatment of experienced HIV-1 patients, renal failure was rare and similar in both arms underscoring the good renal tolerance to etravirine in HIV-pretreated patients. Etravirine in combination with darunavir/ritonavir further widens the choice of antiretroviral therapy in treatment experienced patients with renal disease [71–73]. 

Urinary obstruction and AKI may develop secondary to stones associated with drugs such as sulphadiazine, acyclovir, indinavir, atazanavir, and rarely trimethoprim-sulfamethoxazole, particularly in patients with underlying risk factors [74–76]. In a case report, efavirenz has been associated with minimal change disease from podocyte injury as well as urolithiasis [77]. Proper volume resuscitation may reduce or reverse stone formation, but the drug may need to be discontinued in some cases [78]. Immune reconstitution inflammatory syndrome (IRIS) which commonly occurs within the first three months of starting HAART has also been associated with AKI. Clinicians should include it as a differential diagnosis of nephrotoxic effect of some antiretrovirals [79, 80]. 

 A selected list of drugs associated with acute kidney injury is shown in Table 2.

## 7. Chronic Kidney Disease

The Infectious Disease Society of America recommends that, at the time of HIV diagnosis, all patients should be assessed for evidence of CKD, and, if present, be appropriately staged for kidney disease. The best way to measure the GFR involves administering a foreign substance like inulin or radio-isotopes that the glomeruli will filter completely as waste, without reabsorption by the tubules, and measuring its clearance over time [81, 82]. Unfortunately, these methods are quite expensive and too complex to use outside the research setting. The 24-hour creatinine clearance is also laborious and awkward, but it has been validated in a small study of HIV-positive patients [83].

 Most nephrologists believe that the use of serum creatinine measurement alone is an insensitive measurement of GFR for all patients and other estimation formulas should be used.

In clinical practice, creatinine-based equations, such as the Cockcroft-Gault (CG) equation that estimates creatinine clearance, the Modification of Diet in Renal Disease (MDRD), and Chronic Kidney Disease Epidemiology Collaboration (CKD-EPI) equations, both which estimate GFR, are used to assess kidney function [78, 84]. The choice of the equation to be used to measure renal impairment is still under contention, but the CKD-EPI equation has been reported to be more accurate than the MDRD study equation overall and across most subgroups [84]. The limitation of the Cockcroft equation is that the muscular mass is estimated by age and weight, which can be misleading in some situations including old age and obesity. In one cross-sectional study to determine the best method for estimating the GFR in HIV-infected subjects, isotopic GFR was correlated with 24-hour urine creatinine clearance, cystatin C levels, and 3 creatinine-based equations, the MDRD, CG, and CKD-EPI in 15 patients. Cystatin C showed the strongest correlation with isotopic GFR (*r* = −0.760, *P* = 0.001). When cystatin C was used as the reference variable for all 106 patients, CKD-EPI proved to be superior to the other equations (*r* = −0.671, *P* < 0.001) [85]. However, all these equations have diminished accuracy at estimating GFR above 60 mls/min/1.73 m^2^ [86, 87]. 

Early studies evaluating the accuracy of Cockcroft-Gault equation in HIV patients yielded conflicting results and did not use inulin or radioisotope clearance as gold standards [88, 89]. In Ghana and South Africa, these formulae were not accurate and the adjustments made to the equations to account for race in the MDRD equation made the estimations less reliable [90, 91]. The jury is still out for the best method of estimating GFR in HIV patients as well as those of different ethnicities.

The use of other potential indicators of kidney injury (biomarkers) among HIV-positive individuals like retinol binding protein (RBP), N-acetyl-beta-D-glucosaminidase (NAG), and neutrophil gelatin-associated lipocalin (NGAL) have been studied as indicators of kidney injury. While NGAL indicates glomerular or proximal tubular dysfunction, RBP and NAG may reflect proximal renal tubular dysfunction [92–94]. Patients on tenofovir without evidence of proteinuria by dipstick have been demonstrated to have higher levels of urinary RBG excretion [95]. 

It is often challenging to distinguish antiretroviral-related renal toxicity from either direct effects of HIV-1 on the kidney or from a multitude of non-HIV-related kidney diseases [22]. In many patients, severe GFR decreases may simply be primarily a reflection of acute intercurrent illness rather than ongoing drug nephrotoxicity. The scenario is further complicated by the fact that HIV-AIDS patients are now living longer and are more predisposed to age-related chronic diseases [68]. 

Chronic kidney diseases due to diabetes mellitus, hypertension, renovascular disease, and chronic glomerulonephritis are on the rise, and these contribute to kidney dysfunction, sometimes through drug interactions [96]. These diseases are likely to be missed if they are not actively searched for in the HIV programs. Tenofovir has also been associated with osteoporosis which may have far reaching consequences in patients with preexisting CKD and its antecedent bone-mineral disorders [97]. For patients with bone-mineral disorders, phosphatemia should be analyzed in a fasting patient, and vitamin D should be assessed when interpreting hypophosphatemia. 

## 8. Recommendations for HAART Use in Patients with Kidney Disease

The evaluation of an HIV-infected patient with suspected kidney disease should follow the usual guidelines utilized for non-HIV-infected patients. AKI should be approached with the usual practice of looking for prerenal, renal, and postrenal causes. The common causes of AKI in the HIV-patient should be actively sought and addressed. Before starting antiretroviral treatment, all patients should be screened for kidney disease according to the Infectious Disease Society of America guidelines [78]. For patients to be initiated on drugs known to cause nephrotoxicity, renal function tests should routinely be performed. This may help to prevent development of CKD, which would require further resource utilization [98]. It is also important to remember that many patients with HIV may present with muscle wasting while receiving HAART, which can lower serum creatinine concentration and falsely support the presence of normal kidney function. In such patients, serum creatinine measurement alone is an insensitive measurement of GFR. Conversely, with HAART therapy patients may gain weight, and creatinine may increase without renal injury.

Clinicians should therefore make appropriate adjustments in drug dosage based on the patient's estimated creatinine clearance as calculated by the Cockcroft-Gault equation or estimated GFR (MDRD, CKD-EPI formulae). The selected regimen should be dose adjusted based on the established guidelines using the estimated GFR and stage of kidney disease.

Most NNRTIs, PIs, fusion inhibitors, integrase inhibitors, and CCR5 antagonists do not require dose modification in CKD or ESRD. However, several drugs need special mention because of their increased use and/or demonstrated adverse effects on the kidneys. The usual dosage of TDF for HIV patients without significant renal insufficiency is 300 mg daily, but it requires dose adjustments at creatinine clearance (CrCl) levels below 50 mL/min as indicated in Table 3. 

Combination therapy such as TDF/FTC (300 mg TDF/200 mg FTC) also requires dose adjustments for CrCl of 30–49 mL/min. Most importantly, the TDF/FTC combination pill is not recommended for patients with CrCl below 30 mL/min. Clinicians may therefore opt to prescribe TDF as a separate drug that is renally adjusted, in combination with other HAART regimens. Alternatively, due to the current lack of concrete data, countries with safer alternative regimens can avoid this drug when the GFR drops below 50 mL/min. Along these lines, several studies have demonstrated irreversible renal injury beyond one year [62, 63, 99]. 

There is also significant drug interaction between TDF and DDI. Thus, when coadministered with TDF, it is important to make appropriate reduction in dose adjustments of DDI in patients weighing 60 kilograms or more [78]. Because the potential toxic interactions between TDF and DDI as well as the lack of immunological efficacy, this combination should be avoided.

Indinavir (800 mg twice daily) can cause dysuria, flank pain, renal colic, hematuria, crystalluria, nephrolithiasis, AKI, CKD, and papillary necrosis and has largely been superseded by newer and more efficacious PIs-like darunavir. One could also argue for a switch of the patients on this drug to newer agents due to the high rates of nephrotoxicity and the high daily fluid intake requirements once on this drug [78]. 

Upon HIV diagnosis, patients should be assessed for preexisting kidney disease by use of urinalysis and calculation of estimated GFR; patients with 1+ proteinuria or GFR <60 mL/min/1.73 m^2^ should be referred to a nephrologist. Patients with diabetes must be tested for microalbuminuria (albumin to creatinine ratio 30–300 mg/mg), a range not detected using conventional urine dipsticks. Additional evaluations (e.g., quantification of proteinuria and renal ultrasonography) should be implemented on a case-by-case basis. Patients at high risk of kidney disease (i.e., black patients, patients with CD4+ T-cell count <200 cells/mm^3^, HIV RNA levels >4,000 copies/mL, diabetes, hypertension, or HCV coinfection) should be screened at least annually for subtle changes in renal parameters; patients on TDF may require monitoring every 3 months [78, 100].

A kidney biopsy is recommended for patients with unexplained kidney disease, especially those with heavy proteinuria or reduced GFR, because they are at the greatest risk of ESRD. The kidney biopsy is also very important in confirming the diagnosis of kidney injury, which is often quite diverse and unpredictable in HIV. As already noted above, tenofovir toxicity can also be diagnosed on biopsy [63]. Since kidney biopsies can be performed safely in HIV patients, they should be employed in the proper setting. For example, a single-center study of kidney biopsy safety confirmed that the incidence of complications related to percutaneous ultrasound-guided kidney biopsy was similar between HIV-positive and HIV-negative patients [101]. However, patients with HIV-hepatitis C coinfection were at a higher risk of biopsy-related complications compared with individuals infected with HIV or hepatitis C alone. This biopsy-related increased risk should be explained to individuals who are HIV-HCV coinfected.

Early referral of CKD patients to clinicians skilled in management of kidney disease may improve patient outcomes. Patients who progress to ESRD should be managed with the available modes of renal replacement therapy (RRT) in the country. All modes of RRT should be available for HIV-infected patients with end-stage renal disease. Although HIV-infected patients managed with peritoneal dialysis had worse outcomes in pre-HAART era [102], currently the choice of dialysis modality between hemodialysis and peritoneal dialysis is not a factor in predicting survival, if patients are stable on HAART [103]. 

Patients with HIV and ESRD can receive kidney transplants. Renal transplantation is both safe and effective in HIV patients who meet established criteria. Although rejection rates are higher in these patients, these rejections respond well to therapy. Several drug interactions between HAART and immunosuppressants exist and should be taken into consideration when devising the immunosupression regimens [104]. In a carefully selected population of patients on HAART with CD4+ T-cell counts of at least 200 per cubic millimeter and undetectable plasma HIV type 1 (HIV-1) RNA levels, both patient- and graft-survival rates were high at 1 and 3 years, with no increases in complications associated with HIV infection. However, there was an unexpectedly high rejection rate especially in patients who received antithymocyte globulin induction therapy. The authors expressed a need for better immunotherapy in these patients [105]. There is also concern of drug interactions between protease inhibitors and calcineurin inhibitors, and the degree to which each affects the other has important implications with regard to organ rejection as well as viral suppression [106, 107]. Thus, patients require vigilant monitoring of drug interactions. Some experts have argued for transplant of HIV-infected patients with kidneys from HIV-infected donors following the ground breaking kidney transplant from an HIV-positive donor in South Africa [108]. This has been successful in four patients followed up for 12 months [109]. 

## 9. Conclusion

HAART and other medical therapies for HIV-related infections have been associated with both short- and long-term toxicities including nephrotoxicity. Antiretroviral therapy can contribute to renal dysfunction directly by inducing acute tubular necrosis, acute interstitial nephritis, crystal nephropathy and renal tubular disorders or indirectly via drug interactions. Kidney abnormalities tend to develop in the setting of multiple treatments and cannot be always attributed to a specific drug. Renal function should therefore be monitored on a regular basis in patients with HIV receiving any antiretroviral agent. With the increase in HAART use, clinicians must screen patients for the development of kidney disease especially if the regimen employed increases risk of kidney injury. It is also important that patients with CKD are not denied the best combinations, especially since most drugs can be adjusted based on the estimated GFR. Early detection of risk factors, systematic screening for chronic causes of CKD, and appropriate referrals for kidney disease management should be advocated for improved patient care. 

## Figures and Tables

**Table 1:** Preferred first-line ART in treatment of naive adults and adolescents.

Target Population	Preferred option
Adults and adolescents	AZT or TDF + 3TC or FTC + EFV or NVP
Pregnant women	AZT + 3TC + EFV or NVP
HIV/TB coinfection	AZT or TDF + 3TC or FTC + EFV
HIV/HBV coinfection	TDF + 3TC or FTC + EFV or NVP

Zidovudine (AZT), tenofovir (TDF), lamivudine (3TC), emtricitabine (FTC), nevirapine (NVP), and efavirenz (EFV). Source: WHO, 2010.

**Table 2:** Selective drugs causing AKI in HIV-infected patients.

Drugs	Acute tubular injury (ATI) or AKI	Acute interstitial nephritis	Other associated abnormalities
TMP-SMX (Bactrim)		+++	Hyperkalemia, crystalluria
*β*-lactams		++	
Sulfadiazine		++	Crystalluria, nephrolithiasis
Fluoroquinolones		+	
Rifampin	+	+	Hypokalemia, hypouricemia, hypernatremia, vasculitis
INH		+	Overdose leads to high anion gap metabolic acidosis
NSAIDs	+/−	+	Proteinuria, secondary minimal change disease, papillary necrosis
Dapsone	+/−		Papillary necrosis
Amphotericin B	+++		Hypokalemia, hypomagnesemia, hypernatremia, NDI
Pentamidine	+++		Crystalluria, hyperkalemia
Foscarnet	+++		Hypercalcemia/hypernatremia, Glomerular crystals
Ganciclovir	+		
Acyclovir	+	+/−	Crystalluria
Indinavir, atazanavir		+	Crystalluria, nephrolithiasis
Abacavir		+/−	
Tenofovir	++		Fanconi, NDI

Key: + mild, ++ moderate, +++ severe injury. NDI: nephrogenic diabetes insipidus.

**Table 3:** Dose adjustment for commonly used NRTIs.

Agent	Normal dose	Estimated GFR (creatinine clearance: CrCl)
Zidovudine	300 mg twice a day orally	100 mg thrice a day orally

Lamivudine	150 mg twice a day orally	30–49 mL/min = 150 mg once a day orally 15–29 mL/min = 100 mg once a day orally 5–14 mL/min = 50 mg once a day orally <5 mL/min = 25 mg once a day orally

Stavudine	30 mg twice a day orally	26–50 mL/min = 15 mg twice a day orally <26 mL/min = 15 mg once a day orally

Didanosine	>60 kg: 200 mg twice a day orally <60 kg, 125 mg twice a day orally	30–59 mL/min = 200 mg once a day orally 10–29 mL/min = 150 mg once a day orally <10 mL/min = 100 mg once a day orally 30–59 mL/min = 150 mg once a day orally 10–29 mL/min = 100 mg once a day orally <10 mL/min = 75 mg once a day orally

Tenofovir	300 mg once a day orally	30–49 mL/min = 300 mg every second day 10–29 mL/min = 300 mg every third day <10 mL/min = 300 mg once weekly

(Note: no dose adjustment necessary for abacavir).
